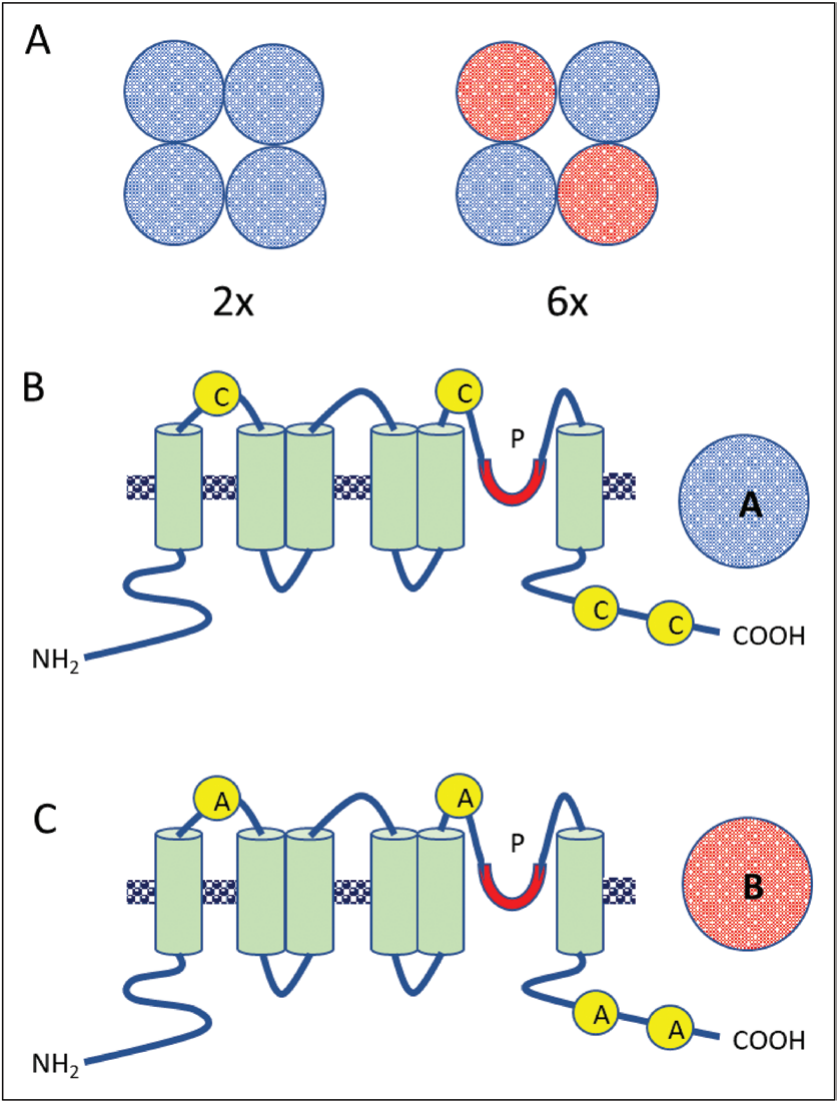# Linking ploidy level with salinity tolerance: NADPH-dependent ‘ROS–Ca^2+^ hub’ in the spotlight

**DOI:** 10.1093/jxb/erz042

**Published:** 2019-02-20

**Authors:** Sergey Shabala

**Affiliations:** 1 International Centre for Environmental Membrane Biology, Foshan University, Foshan, China; 2 Tasmanian Institute of Agriculture, University of Tasmania, Hobart, Australia

**Keywords:** Ca^2+^ transport, H_2_O_2_, K^+^ retention, K^+^/Na^+^ homeostasis, Na^+^ exclusion, polyploid, salinity stress

## Abstract

This article comments on the following paper:

**Liu Y, Yu Y, Sun J, Cao Q, Tang Z, Liu M, Xu T, Ma D, Li Z, Sun J.** 2019. Root-zone-specific sensitivity of K+- and Ca2+-permeable channels to H2O2 determines ion homeostasis in salinized diploid and hexaploid Ipomoea trifida. Journal of Experimental Botany 70, 1389–1405.

**Polyploidy is considered to be a driving force in plant evolution that enabled adaptation to adverse environmental conditions such as soil salinity. This phenomenon is examined by Liu *et al.* (2019) in relation to root-zone-specific ion transport, and can be explained by more efficient operation of an NADPH-dependent ‘ROS–Ca^2+^ hub’ and desensitization of ROS-inducible cation channels in polyploid lines. Two hypotheses include that non-selective cation channels in polyploid lines are formed of chimeric tetramers, with some subunits having modified thiol groups (hence, reduced sensitivity to H_2_O_2_), or alternatively that inactivation of Ca^2+^ channels and higher Ca^2+^-ATPase pump activity may reduce the level of cytosolic free Ca^2+^ and provide a negative control over NADPH oxidase operation.**

Whole-genome duplication, or polyploidy, is considered to be a driving force in plant evolution that enabled better adaptation to some adverse environmental conditions (Adams and Wendel, 2005; Parisod *et al.*, 2010). Polyploid plants demonstrate enhanced tolerance to a range of biotic and abiotic stresses, including soil salinity (Chao *et al.*, 2013). A good example is hexaploid bread wheat (*Triticum aestivum*; genome BBAADD) that is more salt tolerant than its tetraploid wheat progenitor (*T. turgidum*) or durum wheat (*T. durum*) (Munns and James, 2003). Genome duplication improved rice resistance to salt stress (Tu *et al.*, 2014), and citrus tetraploid genotypes are more tolerant of moderate saline stress than diploids (Saleh *et al.*, 2008; Mouhaya *et al.*, 2010). The link between ploidy level and salinity tolerance seems to be reciprocal, with the recent report by Barkla *et al.* (2018) showing that salt treatment led to a significant increase in ploidy levels in the epidermal bladder cells of the halophyte *Mesembryanthemum crystallinum*.

The physiological mechanisms explaining improved salt tolerance with increasing level of ploidy remain obscure. Yang *et al.* (2014) showed superior salinity stress tolerance in a synthetic allohexaploid wheat (neo-6×) compared with its tetraploid (*T. turgidum*; BBAA) and diploid (*Aegilops tauschii*; DD) parents, and attributed this to regulatory transition of the HKT1;5 gene from constitutive high basal expression to induced high expression upon salt stress. However, no HKT1;5 activity was measured, and the only evidence provided was a difference in the xylem Na concentration and minor variations in HKT1;5 expression in leaves at one specific timepoint (with a plethora of other reported differences in gene expression between genotypes).

Recent years have witnessed a paradigm shift towards recognition of plant tissue tolerance (e.g. a capacity of tissues to function while containing a high internal Na^+^ and Cl^–^ concentration; Munns *et al.*, 2016) as a key determinant of overall salinity stress tolerance. Cytosolic K^+^ retention, i.e. an ability of root and mesophyll cells to prevent NaCl-induced K^+^ efflux, has been shown to be an essential component of the tissue tolerance mechanism (Shabala and Pottosin, 2014; Shabala *et al.*, 2016; Wu *et al.*, 2018). Recently Chao *et al.* (2013) analyzed the elemental composition of leaves from 349 Arabidopsis accessions and 89 RILs and reported a strong correlation between the ploidy level and leaf K^+^ content. Can this be an explanation for superior salinity tolerance in polyploids? And if so, how is this trait regulated?

## Root-zone ion transport

Liu *et al.* (2019) conducted a comprehensive study of the relationship between the ploidy level of *Ipomoea trifida* plants and root-zone-specific ion transport under saline conditions. They convincingly showed that superior tolerance of autohexaploid (6×) *I. trifida* as compared with diploid (2×) plants was conferred by reduced sensitivity of plasma membrane K^+^-permeable channels in the meristem root zone and increased sensitivity of Ca^2+^-permeable channels in the elongation and mature root zones to H_2_O_2_. This differential ROS sensitivity confers superior K^+^ retention and Na^+^ exclusion under salt stress, explaining the salt-tolerant phenotype in hexaploid plants. As the reported H_2_O_2_ levels were the same in double- and hexaploid lines, the above difference cannot be attributed to higher activity of antioxidant enzymes and suggests changes in sensitization of ROS-activated ion channels in the root epidermis.

The mechanisms of ion channel activation by ROS are poorly understood. It is generally assumed that the major targets of ROS-induced modification of proteins are reactive cysteine residues (Alansary *et al.*, 2016). A reactive cysteine contains a thiolate group (S-) which reacts with H_2_O_2_ while the thiol groups (SH) do not react physiologically with H_2_O_2_ unless the reaction is catalyzed (Forman *et al.*, 2004). The direct proof for this comes from experiments by Garcia-Mata *et al.* (2010), who used a heterologous expression system to show that the K^+^ outward-rectifying SKOR channel was activated by by H_2_O_2_ via targeted oxidation of Cys168 at the S3 α-helix within the channel’s voltage sensor. Thus, the difference in ROS-induced K^+^ and Ca^2+^ fluxes between 2× and 6× plants in Liu *et al.* (2019) may potentially be explained by desensitization of the appropriate transport system to H_2_O_2_ resulting from modification of thiol groups in the sensory domain.

## A ‘ROS–Ca^2+^ hub’

Another important observation by Liu *et al.* (2019) was that the magnitude of NaCl-induced K^+^ efflux in the diploid line was reduced by twofold in plants treated with DPI, a known inhibitor of NADPH oxidase. NADPH oxidase is a plasma-membrane-bound enzyme complex from the NOX family, which faces the extracellular space (Marino *et al.,* 2012). Discovered first as part of the plant hypersensitive (HR) response to pathogens, this enzyme has recently emerged as a critical component of stress signaling mechanisms in response to a broad range of abiotic stresses, including salinity (Miller *et al.*, 2010; Ma *et al.*, 2012; Shabala *et al.*, 2015). NADPH oxidase can stabilize SOS1 transcripts (Chung *et al.*, 2008), thus assisting plants in reducing the salt load, and is involved in generating the stress-induced Ca^2+^ ‘signatures’ that mediate rapid systemic signalling (Miller *et al.*, 2010).

The concept of a ROS–Ca^2+^ hub was recently put forward (Demidchik and Shabala, 2018; Demidchik *et al.*, 2018) and implies that Ca^2+^-activated NADPH oxidases work in concert with ROS-activated Ca^2+^-permeable cation channels to generate and amplify stress-induced Ca^2+^ and ROS signals (Box 1). Interestingly, an effect of DPI on K^+^ fluxes was not observed in the 6× line (Liu *et al.*, 2019), suggesting that NADPH oxidase was already inactivated in the polyploid. This inactivation may be a result of either decreased NADPH oxidase phosphorylation by BIK1 (Kadota *et al.*, 2014; Box 1) or low activity of Rac/Rop GTPases (Baxter-Burrell *et al.*, 2002). More active Ca^2+^-ATPase activity in a hexaploid line or inactivation of Ca^2+^ channels resulting from its interaction with CaM (DeFalco *et al.*, 2016) or decreased CDPK-catalyzed phosphorylation (Zhou *et al.*, 2014) may also be the reason for ROS–Ca^2+^ hub activity ceasing (Box 1).

**Box 1 A tentative model for the operation of an NADPH-dependent ‘ROS–Ca^2+^ hub’ in diploid and hexaploid lines**

In the 2× line, apoplastic H_2_O_2_ produced by NADPH oxidase stimulates Ca^2+^ uptake through non-selective cation channels (CNGC in the model) and forms a positive feedback loop, resulting in an avalanche-like increase in cytosolic free Ca^2+^. Because of the massive Ca^2+^ influx into the cell, the plasma membrane is depolarized, triggering K^+^ efflux through the GORK channel. NADPH oxidase operation requires the phosphorylation of one of its terminal domains, mediated by BIK1 (Kadota *et al.*, 2014). Operation of CNGC is also dependent on binding of calmodulin (CaM) to the IQ motif in the C terminus (DeFalco *et al.*, 2016). In a hexaploid line, inactivation of Ca^2+^ channels resulting from its interaction with CaM (DeFalco *et al.*, 2016) or decreased CDPK-catalyzed phosphorylation (Zhou *et al.*, 2014) reduce NADPH oxidase activity. Higher Ca^2+^-ATPase pump activity also reduces the level of cytosolic free Ca^2+^ and provides a negative control over NADPH oxidase operation. BIK1, the plasma-membrane-associated kinase; CPK, calcium-dependent protein kinase; NT, a putative CaM-binding motif; DPZ, depolarization; CaM, calmodulin; IQ, a conserved isoleucine–glutamine motif in the C terminus.

**Figure:**
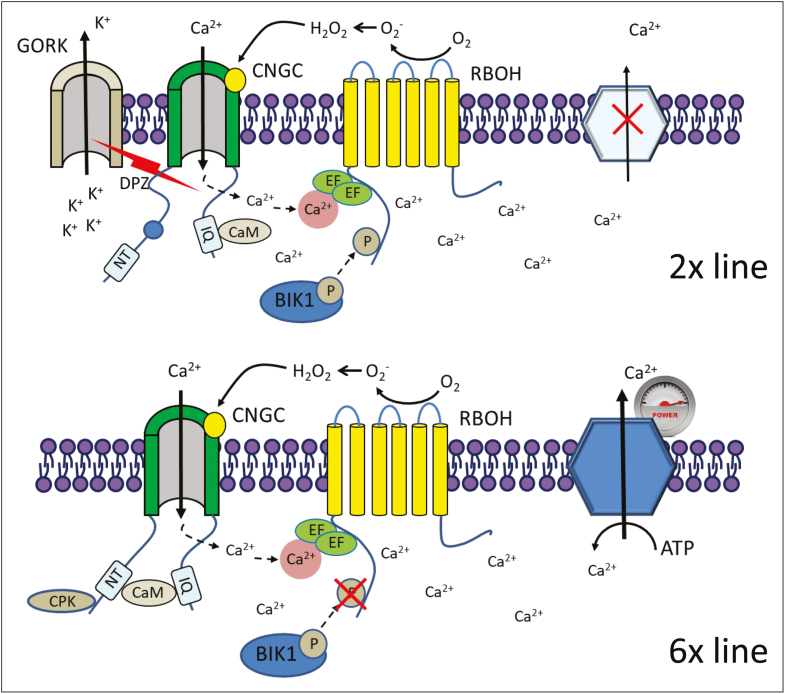

Contrary to animal systems, plant genomes do not encode any Ca^2+^-selective ion channels (Demidchik *et al.*, 2018), with Ca^2+^ transport across the plasma membrane mediated by non-selective cation channels (NSCCs). While the genetic origin of NSCCs remains unknown, two major classes – CNGCs (cyclic nucleotide-gated channels) and GLRs (glutamate receptors) – are known in Arabidopsis (with 20 members in each class; Maser et al., 2001). NSCCs can be activated by ROS (Demidchik *et al.*, 2018). GLRs are believed to be tetramers consisting of different subunits (Price *et al.*, 2013). CNGCs can also form chimeric channels (Zhong *et al*., 2003), and plants harbouring the ATCNGC11/12 gene showed a phenotype with constitutively activated (ROS-burst-related) defence responses to pathogens (Yoshioka *et al.*, 2006). Keeping this in mind, one may hypothesize that polyploid lines may encode chimeric NSCCs with altered ligand-gated properties and reduced sensitivity to H_2_O_2_ (Box 2). It was shown that replacement of the positively charged lysine (Lys1110) with the neutrally charged asparagine (K1110N) or the negatively charged amino acid glutamic acid (K1110E) in the mammalian TRPM2 channel generated mutants that failed to induce an increase in free cytosolic calcium concentration in response to H_2_O_2_ (Kim *et al.*, 2013). It remains to be shown if the similar substitution of one or several amino acids in chimeric NSCCs may desensitize them, thus altering ROS–Ca^2+^ hub operation kinetics and affecting plant salt stress signaling and ionic homeostasis, explaining salt-tolerant phenotype in polyploid lines.

**Box 2 Suggested model explaining the desensitization of cation channels in polyploid lines by chimeric protein assembly**

The model assumes that Ca^2+^ and K^+^ fluxes across the plasma membrane are mediated by cyclic nucleotide-gated channels (CNGCs). Such CNGCs are made up of four subunits, each having one pore region and six transmembrane domains (Demidchik and Shabala, 2018). In a diploid (2×) line, all subunits are identical (panel A; blue) and harbour cysteine (C in the model) residues in both external and enteral loops (panel B) and, thus, can be activated by H_2_O_2_ from either the apoplastic or the cytosolic side. In a hexaploid line (6×), two out of four units have cysteine replaced by the neutrally charged asparagine (A in the model; panel C). The chimeric channel is formed of two type A (blue) and two type B (red) subunits with cysteine substituted by asparagine (or with some other non-ROS-binding amino acid). Such a chimeric channel has fewer ligand (H_2_O_2_)-binding sites and thus reduced sensitivity to ROS. P, pore.

**Figure:**